# Organizing Pneumonia Preceding Rheumatoid Arthritis

**DOI:** 10.1155/2014/758619

**Published:** 2014-01-28

**Authors:** Yoshiaki Kinoshita, Atsuhiko Sakamoto, Kouko Hidaka

**Affiliations:** Division of Respiratory Medicine, Department of Internal Medicine, National Hospital Organization, Kokura Medical Center, 10-1 Harugaoka, Kokura-minamiku, Kitakyushu 802-8533, Japan

## Abstract

Rheumatoid arthritis patients are susceptible to interstitial lung disease, and joint manifestations of rheumatoid arthritis usually precede lung involvements by several years. Organizing pneumonia, as the first manifestation of rheumatoid arthritis, is extremely rare, and its clinical features remain currently unknown. We present a case and a literature review of patients who were pathologically diagnosed with organizing pneumonia first and met the diagnostic criteria of rheumatoid arthritis later. In this review, we observed the following: (1) patients with organizing pneumonia preceding rheumatoid arthritis have a high prevalence of rheumatoid factor or anticyclic citrullinated peptide antibodies; (2) almost all patients developed rheumatoid arthritis within one year after the diagnosis of organizing pneumonia. We suggest that patients with organizing pneumonia and positive for either rheumatoid factor or anticyclic citrullinated peptide antibody should be cautiously followed up regarding the development of rheumatoid arthritis, particularly during the first year after the diagnosis of organizing pneumonia.

## 1. Introduction

Patients with a connective tissue disorder (CTD) are susceptible to lung involvement and some histopathological patterns of interstitial lung disease (ILD), including usual interstitial pneumonia (UIP), nonspecific interstitial pneumonia (NSIP), organizing pneumonia (OP), and diffuse alveolar damage, which occasionally occur in rheumatoid arthritis (RA) [1–4]. Joint manifestations of RA usually precede lung involvements by several years; however, in less than 10% of cases of RA associated UIP or NSIP, ILD may be the initial manifestation of RA [5, 6]. Alternatively, OP as the initial manifestation of RA is extremely rare, and its clinical features remain unknown. Here we present a case and a literature review of patients who were pathologically diagnosed with OP and met the diagnostic criteria of RA later.

## 2. Case Report

**Figure 1:**
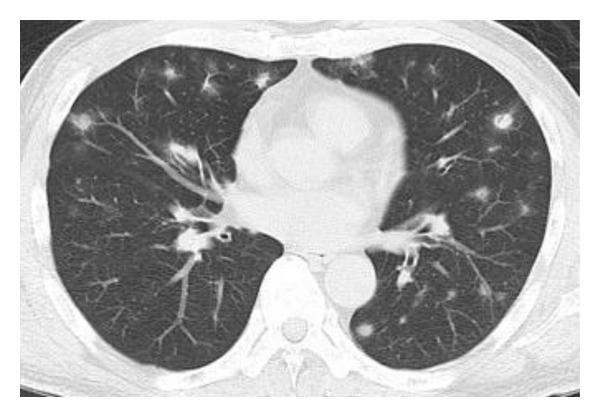
A chest computed tomography showed bilateral multiple nodules predominantly subpleural parenchyma.

**Figure 2:**
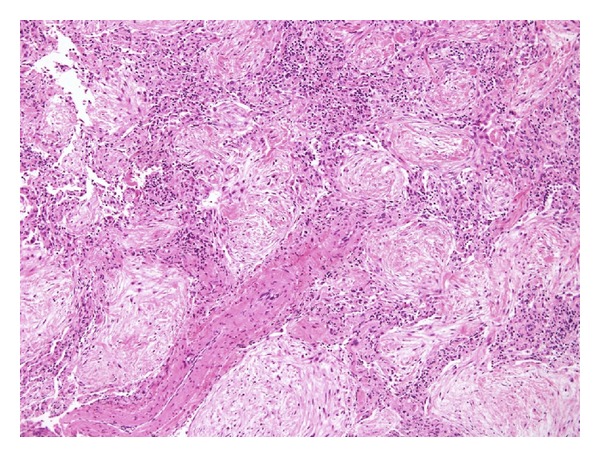
Microscopic examination revealed inflamed granulation tissue occupying terminal bronchioles and alveolar space (hematoxylin-eosin; original magnification, ×40).

A 58-year-old male with a history of hypertension visited our hospital with nonproductive cough, dyspnea, weight loss, and fever. He had a smoking history of 2 pack cigarettes/day but had stopped smoking since a year. His laboratory results were as follows: leukocytes, 6800/*μ*L; C-reactive protein, <0.03 mg/dL; KL-6, 264 U/mL (normal value, <500 U/mL); antinuclear antibody, <40; rheumatoid factor (RF), 69 U/mL (normal value, <15 U/mL); and anticyclic citrullinated peptide antibody (CCP), 50.9 U/mL (normal value, <4.5 U/mL). Chest computed tomography revealed bilateral multiple nodules, predominantly in the subpleural parenchyma (Figure 1). We performed a surgical lung biopsy; microscopic examination revealed inflamed granulation tissue occupying terminal bronchioles and alveolar space, consistent with OP features (Figure 2).

Oral prednisone (40 mg/day) therapy was initiated and considering the dramatic improvement, tapering of the dose was possible. Oral prednisone was discontinued after 16 months, and the patient was followed up without medication. Two years after onset, he complained of diffuse arthralgia that persisted for a month, and his serum concentrations of RF and CCP were markedly increased (RF, 525 U/mL; CCP, 226 U/mL). He met the 2010 ACR/EULAR criteria for the classification of RA. Therefore, oral salazosulfapyridine (500 mg/day) therapy was prescribed, and diffuse arthralgia gradually improved.

## 3. Discussion

Using PubMed search, we performed a literature review of patients who were pathologically diagnosed with OP and met the diagnostic criteria of RA later. Including our case, seven cases have been reported in the literature (Table 1). Seven patients comprised four females and three males, with a median age of 64 years (range, 33–86 years). At the diagnosis of OP, RF was examined in six cases (86%) and CCP in three cases (57%), of which RF was positive in five cases (83%) and CCP in all cases (100%), respectively. The mean time interval from prodromal respiratory symptoms to joint symptoms was 7.8 months (range, 0.5–24 months). All patients were treated with prednisone and responded well; only one patient had a relapse of OP.

It has been quite difficult to identify underlying ILD diseases among patients who have clinical features implying CTDs without meeting current rheumatologic criteria. Therefore, several classifications emphasizing autoantibody results have been proposed, including undifferentiated CTD [1], autoimmune featured-ILD [2], and lung-dominant CTD [3]. However, in so-called “occult CTDs,” the clinical significance of these classifications and evidence for the usefulness of autoantibodies have not yet been established. Meanwhile, in this review, we found that patients with OP preceding RA have a greater prevalence of RF or CCP compared with previous reviews of patients with cryptogenic and secondary OP (RF, 3.4–19.7%; CCP, 4.2%) [12–14]. Early identification of RA is becoming increasingly important because early therapy has been reported to preserve joint function [15]. Therefore, patients with OP, who are positive for RF or CCP antibodies, should be cautiously followed up regarding the development of RA.

The present review indicates that the time interval from prodromal respiratory symptoms to joint manifestations is within one year in 6/7 (86%) cases. Therefore, we recommend to cautiously follow up the development of RA, particularly during the first year after the diagnosis of OP. However, in the present case, the interval was longer and reached 24 months; this delay may be attributed to the prolonged steroid therapy (16 months). Steroid therapy is effective in OP, and the treatment duration is usually between six and 12 months; however, it can considerably vary depending on clinical situations, particularly because of the frequent relapse after discontinuation of therapy [16, 17]. Corticosteroids have anti-inflammatory effects and show efficiency in joint manifestations of RA. Prolonged corticosteroid use may delay the appearance of joint symptoms in RA; therefore, a longer observation may be suitable for such cases.

## Figures and Tables

**Table 1:** Characteristics and outcome of seven patients reported in literatures.

Reference	Sex, Age	Autoantibodies		Therapy (PSL)	Interval^†^, mo	Response/Relapse
		RF*	CCP			
[7]	F, 69	N.A.	N.A.	1 mg/kg	8	Improved/Yes
[7]	F, 33	Positive	N.A.	40 mg	3	Improved/No
[8]	F, 68	1 : 640	N.A.	60 mg	0.5	Improved/No
[9]	F, 86	1 : 160	50.8 U/mL	pulse only	11	Improved/No
[10]	M, 71	159 U/mL	>100 U/mL	30 mg	0.6	Improved/No
[11]	M, 65	Negative	N.A.	80 mg	6	Partial response/No
Present case	M, 58	69 U/mL	50.5 U/mL	40 mg	24	Improved/No

RF: rheumatoid factor, CCP: anticyclic citrullinated peptide antibody, N.A.: not available, PSL: prednisone.

*RF by latex fixation (positive/negative), the concentration of rheumatoid arthritis particle agglutination (normal value, <1 : 40), or quantitation of RF (normal value, <15 U/mL).

^†^The interval from prodromal respiratory symptoms to joint manifestations.
